# Ozone Sensing by In_2_O_3_ Films Modified with Rh: Dimension Effect

**DOI:** 10.3390/s21051886

**Published:** 2021-03-08

**Authors:** Ghenadii Korotcenkov, Vaclav Nehasil

**Affiliations:** 1 Department of Theoretical Physics, State University of Moldova, MD 2009 Chisinau, Moldova; 2 Department of Surface and Plasma Science, Charles University, CZ-18000 Prague, Czech Republic; vaclav.nehasil@mff.cuni.cz

**Keywords:** surface modification, atomic dispersion, clustering, conductometric response, adsorption, decomposition, modeling

## Abstract

We considered the effect of coverage of the surface of In_2_O_3_ films with rhodium on the sensitivity of their electrophysical properties to ozone (1 ppm). The surface coverage with rhodium varied in the range of 0–0.1 ML. The In_2_O_3_ films deposited by spray pyrolysis had a thickness of 40–50 nm. The sensor response to ozone depends on the degree of rhodium coverage. This dependence has a pronounced maximum at a coverage of ~0.01 ML of Rh. An explanation is given for this effect. It is concluded that the observed changes are associated with the transition from the atomically dispersed state of rhodium to a 3D cluster state.

## 1. Introduction

Rhodium (Rh) is one of the noble metals (Pt, Pd, Au) widely used for surface modification of metal oxides designed for gas sensors [1,2,3] and catalysts [4,5,6]. In many reactions, rhodium exhibits specific properties that are not characteristic of other noble metals. Since it gives hope for the development of gas sensors and catalysts with improved parameters, it is of great interest in science and technology. Studies of the catalytic properties of Rh-based catalysts confirmed these expectations. It has been shown that Rh-based catalysts are promising for oxidation of carbon monoxide, catalytic carbonylation of methanol to produce acetic acid, in the reduction of nitrogen oxides to nitrogen and oxygen [4,5,6] and so on. It is believed that the high activity of rhodium is associated with its p-semiconducting properties during oxidation and with a high population of oxygen species that are weakly bound to the surface [7,8]. At the time, it was found that supported rhodium exhibits dispersion-dependent catalytic activity [4,9,10]. As for gas sensors, we do not have a sufficient experimental base for any specific conclusions regarding the prospects for using rhodium to optimize the gas-sensitive characteristics of metal oxide sensors. We also do not have generally accepted views on the mechanism of the influence of rhodium on the parameters of the sensor. We also do not know how important the size effect is for rhodium-modified sensors. The results obtained in the study of the properties of gas sensors based on metal oxides modified with rhodium are too ambiguous [3,11,12].

Recently, interest in catalysts based on atomically dispersed or single-atom noble metals has significantly increased [5,13,14,15]. It is believed that the atomic dispersion generates high uniformity in the metal distribution over the catalyst surface and allows using up to 100% of atoms as active centers [16]. Under certain conditions, atomically dispersed noble metals, including Rh, exhibited significantly higher activity in comparison with bulk material and clusters. It was found that it is due to unique electronic structure and unsaturated coordination environments [15,17]. It is important to note that a number of studies [18,19,20] have shown that the unusual properties of atomically dispersed noble metals should also be taken into account when considering the gas-sensitive characteristics of metal oxide gas sensors.

Earlier, in [21], we found that Rh deposited on the surface of In_2_O_3_ films at a coverage less than 0.01 ML is in an atomically dispersed state or close to it. On the other hand, it begins to form 3D clusters at a coverage of more than 0.01 ML. In this regard, we decided to evaluate the effect of this process on the sensor response of In_2_O_3_ films modified with rhodium to ozone. Ozone is a strong oxidant [22], promising for use in environmentally friendly technologies, such as cleaning of potable water and soil, disinfection of plant and animal products, textile bleaching, the treatment of swimming pools and contaminated gases, complete oxidation of waste gases from the production of various organic chemicals, and sterilization of medical supplies [23]. However, the presence of ozone in ground atmospheric layers is harmful to human health, as ozone is highly toxic above concentrations of 0.1 mg/m^3^ [24]. This makes it necessary to control its concentration in the surrounding atmosphere [25]. It has been shown in numerous works that In_2_O_3_ has significant advantages for the development of sensors aimed for this purpose [26,27,28,29]. In_2_O_3_ is also widely used as a model material for studying various gas-sensing effects [29,30,31,32,33,34]. In addition, according to [16,35], metal oxides such as In_2_O_3_ with a high concentration of oxygen vacancies are excellent support for isolated metal atoms. It was established that surface oxygen vacancies in metal oxide play the role of “traps’’ that accommodate and stabilize isolated metal atoms.

It should be noted that our studies and conclusions refer to surface-modified metal oxides. During bulk doping, carried out during synthesis or long-term high-temperature annealing, many additional factors arise [36], which significantly complicate the understanding and interpretation of the results obtained.

## 2. Materials and Methods

The In_2_O_3_ films were deposited by spray pyrolysis on the alumina substrate using the technology described in [37]. The In_2_O_3_ films used in these studies were deposited at temperatures of 350–375 °C and 450–475 °C. For convenience, from now on these films will be designated as LT (low temperature) and HT (high temperature) films, respectively. The In_2_O_3_ films were 40–50 nm thick. Earlier in [28,29] we showed that such thicknesses are optimal for the development of sensors designed to detect oxidizing gases such as ozone (Figure S1b, supplementary materials). In this case, it is possible to exclude the influence of the gas diffusion on the sensor response, which is very important for active gases like ozone. The structural parameters of such films can be found in [38]. According to Atomic Force Microscopy (AFM) measurements, the crystallites forming the LT and HT films had an average size of 8–12 nm and 13–17 nm, respectively. Typical AFM image and X-ray Diffraction (XRD) patterns of In_2_O_3_ films used are shown in Figure S2 (supplementary materials).

Rhodium (Rh) was deposited to the surface of the films using a microelectron beam evaporation source described in [39]. Taking into account the absence of continuous rhodium coating of the In_2_O_3_ surface, the degree of rhodium coverage was estimated in ML. According to our estimation, average thickness of 0.03 nm corresponded to approximately 0.1 ML of Rh. The thickness of the applied rhodium was controlled by the deposition time. Deposition rate was determined by an oscillating quartz microbalance system (MTM-10, Tectra GmbH, Frankfurt, Germany) and was ~0.025–0.03 nm/min. This means that the evaporation for 60 s allowed the formation of a coating corresponding to 0.1 ML of Rh.

Rh/In_2_O_3_-based sensors were tested in a flow-through measuring cell. The cell volume was ~0.3 cm^−3^, which made it possible to control gas-sensitive effects with time constants greater than 3–5 s. The test gas had a concentration of 1 ppm. The ozone source was developed based on a UV lamp. The air humidity was maintained at 45–50% RH. The operating temperature of the sensors varied in the range of 25–450 °C. It is important to note that the structural properties of In_2_O_3_ films were stable during these measurements. Previous studies, the results of which were published in [38], did not reveal any changes in the morphology and crystallite size of such films up to annealing temperatures of 550–600 °C, which could be detected by SEM images and XRD measurements. As a result of measuring gas sensing characteristics, it was found that the maximum sensitivity to ozone was observed at temperatures of 200–250 °C (Figure S1, supplementary materials), regardless of the conditions used for surface modification of In_2_O_3_ films with rhodium. Therefore, the main measurements were carried out at a temperature of 250 °C. The sensor response was calculated as the ratio of the film resistance measured in an atmosphere containing ozone and in atmosphere without ozone (S = R_ozone_/R_air_). Time constants of transient processes (response and recovery time) were determined on the 0.9 level from steady state value of film conductivity. The measurements were carried out after artificial aging of the sensors, which involved several preliminary testing procedures for reducing and oxidizing gases at a temperature of 400 °C. Gold contacts to the films were formed by vacuum deposition. The distance between the contacts was ~2 mm, which significantly reduced the possible influence of the contacts on the sensor signal.

## 3. Results and Discussions

**Figure 1:**
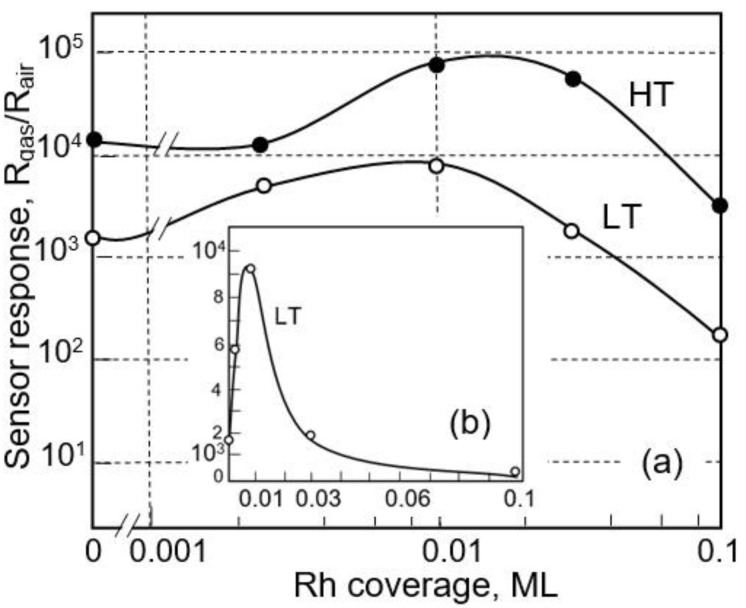
Effect of surface modification of LT and HT In_2_O_3_ films with rhodium on their conductometric response to ozone (~1 ppm): data are presented on (**a**) logarithmic and (**b**) linear scales (*T*_oper_ = 250 °C).

**Figure 2:**
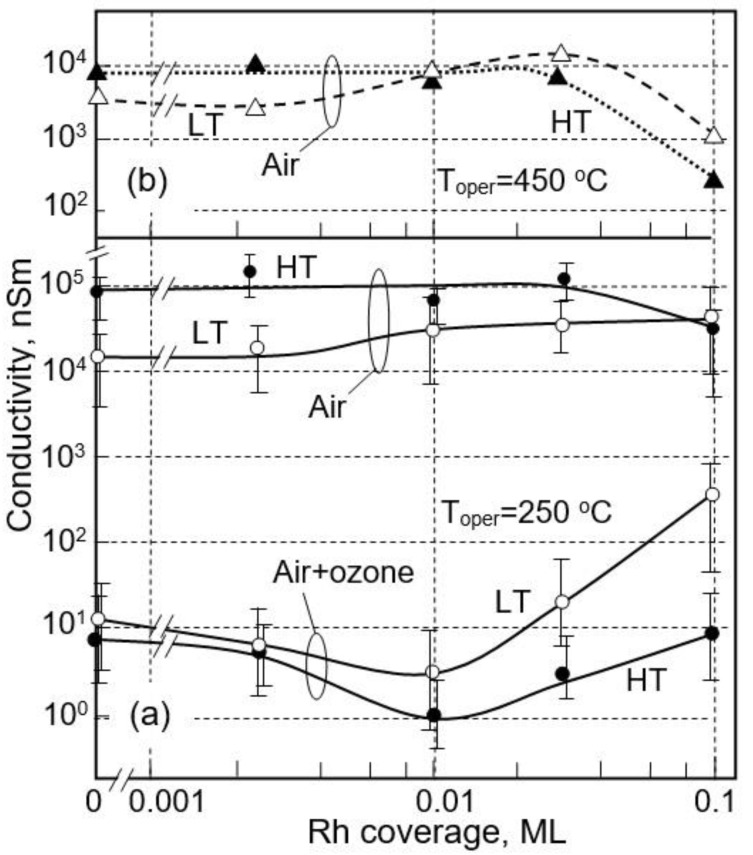
Change in the conductivity of LT and HT of In_2_O_3_ films after surface modification with rhodium, measured in an atmosphere of air and ozone at a temperature (**a**) 250 °C and (**b**) 450 °C.

It is important to note that the gas-sensitive characteristics of undoped In_2_O_3_ films, including the sensor response to ozone, are described in sufficient detail in [28,29,31,34,40,41,42,43]. When studying films modified with rhodium, we found that the conductometric response of these films to ozone obeys the general laws established for undoped films (Figure S1, supplementary materials). Therefore, in this article, we will only consider the effect of surface modification with rhodium on the absolute values of the sensor response to ozone and the time of this response. The results of this consideration are presented in Figure 1 and Figure 2. The data shown in these figures were obtained by averaging results of five measurements taken on two identically prepared samples. In particular, Figure 1 demonstrates the effect of surface modification with rhodium on the response of In_2_O_3_ based conductometric gas sensors, while Figure 2 shows the effect of surface modification on the conductivity of In_2_O_3_ films measured in an air atmosphere with and without ozone.

When analyzing the behavior of the sensor response of rhodium-modified In_2_O_3_ films, the following important features of the effect of rhodium can be distinguished:

1. Rhodium modification has a noticeable effect on sensor response. The sensor response of rhodium-modified In_2_O_3_ films has a clearly pronounced maximum at Rh coverage of ~0.01 ML. As the coverage increases further, the sensor response decreases. This suggests that the activity of rhodium on the In_2_O_3_ surface depends on the degree of surface coverage with Rh. At a low degree of coverage of the In_2_O_3_ surface, rhodium acts as an activator of the interaction of ozone with the surface of In_2_O_3_. On the other hand, at a larger degree of coverage it behaves as a poisoner or a catalytically active filter that prevents the interaction of ozone with In_2_O_3_. Previously, we observed this effect after surface modification of the SnO_2_ surface with silver and palladium [44].
2. The effect of surface modification with rhodium on the gas-sensing characteristics of In_2_O_3_ films depends on the initial properties of the film used. In particular, In_2_O_3_ films deposited at higher temperatures had a higher sensitivity to ozone. However, it should be noted that this behavior is typical for sensors deposited by spray pyrolysis. In particular, we discussed this effect and the reason for this behavior earlier in the articles [29,40]. For us, it is more important that the nature of the effect of surface modification with rhodium on the sensor characteristics does not depend on the deposition temperature of the In_2_O_3_ films, which increases the reliability of the results obtained.
3. Comparison of the obtained results with the X-ray photoelectron spectroscopy (XPS) measurement data presented in [21] shows their clear correlation. The decrease in the sensor response occurs at the Rh coverage greater than 0.01–0.02 ML. As was established in [21], at this coverage, Rh3d_5/2_ binding energy begins to decrease (see Figure S3, supplementary materials). When analyzing XPS data [21], we concluded that a decrease in binding energy is a consequence of an increase in the size of rhodium nanoparticles. As we mentioned above, if rhodium on the surface of In_2_O_3_ is in the atomic state, or close to this state, at a coverage of less than 0.01 ML, then when the Rh coverage exceeds 0.01 ML, the formation of 3D clusters begins. The size of these 3D clusters increases with increasing coverage of the In_2_O_3_ surface with rhodium and can reach 1–2 nm with coatings 0.1 ML of Rh [21].

Taking into account the above processes occurring on the surface of In_2_O_3_ with an increase in the degree of coverage with rhodium, one can conclude that the size-effect really plays an important role in changing the activity of rhodium used to modify the surface of gas-sensitive materials. If the atomically dispersed state of rhodium promotes an increase in the sensitivity to ozone, then the transition to the cluster state is accompanied by the drop in the sensor response. Moreover, with a certain degree of coverage with Rh, the sensor response becomes even less than the sensor response observed for unmodified In_2_O_3_ (see Figure 1). This explains why, when using the same metal Rh to modify the surface of metal oxides, in some cases one can observe an increase [3], and in others, a decrease in the sensor response to oxidizing gases [12,45,46]. It can be assumed that, in all likelihood, Staerz et al. [12,46], who observed reduction in sensor response to oxidizing gases after surface modification with Rh, have used too high a concentration of Rh. Another reason may be related to the difference in surface modification methods. Typically, the chemical methods used in [12,46] tend to form agglomerates on the surface of a solid rather than individual atoms [14]. Indeed, scanning transmission electron microscopy of studied samples has shown that rhodium on the surface of metal oxides was in the form of Rh_2_O_3_ particles and clusters with the size about 1–3 nm [12]. This is in good agreement with the results reported by Matsubu et al. [47], who demonstrated that isolated Rh atoms and Rh nanoparticles on the same support can exhibit uniquely different catalytic activity and selectivity. This situation also applies to other noble metals. For example, Koziej et al. [48] noted that Pd on the surface of SnO_2_ presented in atomic or nearly atomic distribution and in the form of palladium oxide particles exhibits different redox properties.

It is important to note here that the observed changes in sensor response when the coverage with Rh exceeds 0.01 ML cannot be attributed to a change in the chemical state of rhodium, the main mechanism responsible for conductivity response of In_2_O_3_–based sensors, modified with rhodium clusters, to reducing gases such as H_2_ or CO [48,49]. This mechanism involves the reduction of rhodium oxide in the atmosphere of reducing gas and its reoxidation in an oxygen-containing atmosphere. Experimentally, this effect has been observed many times [50,51,52]. However, according to numerous studies [12,46,53,54], rhodium clusters on the surface of metal oxides in an oxygen atmosphere, especially at elevated temperatures, are already in an oxidized state. For example, Padeste et al. [51] established that Rh clusters were oxidized to Rh_2_O_3_ at 200 °C. Schiinemann et al. [55] reported that after calcination of the Rh/NaY catalyst to 380 °C both RhO_2_ and Rh_2_O_3_ were identified on the surface of zeolite support. Hwang et al. [56] and Hülsey et al. [52] have shown that the highly dispersed rhodium clusters have been fully reoxidized to bulk oxide even at room temperature. Hämäläinen [57] also deposited rhodium films and found that deposition of Rh in an ozone atmosphere at T < 200 °C leads to the formation of Rh_2_O_3_ films. This means that the Rh^3+^ state is the most probable rhodium state in a Rh cluster in an oxygen atmosphere at T < 300 °C. As for a possible change in the charge state of Rh upon interaction with ozone, if it occurs (Rh^3+^ ↔ Rh^4+^) during the transition from one oxide state to another (RhO_2_ ↔ Rh_2_O_3_), then it should not affect the sensor response. It is known that Rh_2_O_3_ and RhO_2_ are stable in an oxygen atmosphere [58]. This means that the RhO_2_ ↔ Rh_2_O_3_ transition under the action of ozone is irreversible. Therefore, if we do not use reducing gases during our experiments, then this transition, RhO_2_ ↔ Rh_2_O_3_, should be observed only one time after the first contact of the Rh/In_2_O_3_ film with ozone, and during subsequent interactions with ozone it should not appear.

If we consider the effect of In_2_O_3_ surface modification with Rh on the film conductivity measured in different atmospheres, we will see that after surface modification with rhodium, changes in the conductivity of the films are insignificant at measurement carried out in air without ozone (Figure 2). When studying the gas-sensitive properties of SnO_2_ films modified with Rh, we observed the same situation [3]. This means that despite the common opinion [59,60] that Rh acts on the surface of metal oxides as a porthole of the oxygen spillover, the dissociation of molecular oxygen into atomic rhodium is not a factor, determining the conductivity of In_2_O_3_ in an oxygen atmosphere at a temperature of 200–250 °C. This behavior is understandable in principle. First, atomic rhodium, unlike cluster rhodium, cannot be a porthole for an oxygen spillover, since for the dissociation of oxygen on the Rh surface two adjacent vacant sites are needed, which atomic rhodium cannot provide. Secondly, the temperature of 250 °C may not be sufficient to activate the dissociation of oxygen on the oxidized surface of rhodium clusters, spillover on In_2_O_3_, and the subsequent diffusion of atomic oxygen on the In_2_O_3_ surface. These assumptions seem to be reliable. Measurement of conductivity at *T* = 450 °C showed that at these temperatures a significant decrease in conductivity in an oxygen atmosphere is indeed observed (see Figure 2). However, this decrease occurs only when the coverage with Rh exceeds 0.03 ML (cluster zone), while in the range of 0.0–0.01 ML of Rh (atomic dispersion), the conductivity practically does not change. This means that atomic rhodium, unlike cluster rhodium, does not really stimulate the dissociative adsorption of oxygen.

The same conclusion cannot be drawn regarding dissociative adsorption of ozone, since the most significant changes in conductivity are observed during conductivity measurement in an atmosphere containing ozone (see Figure 2). Namely, in the region of the atomically dispersed state of rhodium, an increase in the resistance of Rh/In_2_O_3_ films is observed, and in the region of the cluster state of Rh, a sharp drop in the resistance of Rh/In_2_O_3_ film occurs. As is known, the conductivity of polycrystalline semiconductor metal oxide films of n-type of conductivity is controlled by the concentration of electrons captured by particles adsorbed on the surface of metal oxide crystallites [61,62]. A decrease in the negative charge leads to an increase in the conductivity of the films, and an increase in the negative charge leads to a decrease in the conductivity. This means that if at low coverages the appearance of rhodium on the In_2_O_3_ surface contributes to the growth of a negative charge on the In_2_O_3_ surface during ozone detection, then this does not happen at high coverages of Rh. Moreover, this charge becomes even less than on the surface of the unmodified metal oxide. Based on the observed regularities, we can assume that the adsorption of ozone on rhodium, which is in an atomic state or close to the atomic state, is accompanied by electron exchange between the metal oxide and chemisorbed oxygen generated due to dissociative adsorption of ozone, initiated by atomic rhodium. This process can be represented as the formation of ozonide ion radical $O_{3}^{-}$ and its subsequent dissociation into atomic and diatomic oxygen species (Equation (1)).

(1) $$O_{3}\underset{{\mathrm{Rh}}^{n+}}{\to }O_{3}^{-}\to O^{-}+O_{2}↑$$

**Figure 3:**
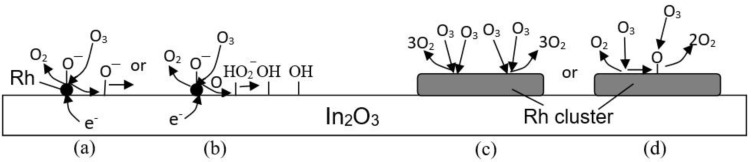
Schematic representation of the processes occurring on the surface of Rh/In_2_O_3_ during ozone detection. (**a**,**b**)—processes occurring on atomic rhodium, (**c**,**d**)—processes on Rh clusters.

Thus, the observed change in the conductivity of Rh/In_2_O_3_ films under the influence of rhodium, measured in an ozone atmosphere, can be explained. The increase in the resistance of the metal oxide when interacting with ozone takes place due to the increase in the concentration of ionized oxygen species on the surface of the metal oxide [34], which occurs due to the appearance of additional centers of dissociative adsorption of ozone, the role of which is played by rhodium atoms (Figure 3a). Such new sites of ozone dissociative adsorption may affect the distribution of surface negative charge, concentrating ionized oxygen species in the local area in proximity around rhodium atoms. Taking into account the configuration of ozone, one could assume that two isolated rhodium atoms located at a distance of less than 0.5–0.7 nm from each other can also participate in the dissociative adsorption of ozone. For information, for 0.01 ML of Rh, the average distance between atoms is ~3 nm. As a result of dissociation, atomic oxygen remains on one of these atoms, which is active for the sensor effect, and molecular oxygen on the other. However, the number of such pairs is naturally much less than the total number of rhodium atoms, and therefore their contribution to the sensor response should be minimal.

Taking into account what is mentioned above, the appearance of a maximum in the dependence of the sensor signal on the degree of coverage of the In_2_O_3_ surface with rhodium in the range of 0.0–0.01 ML is a simple consequence of an increase in the number of rhodium atoms. If we take into account that one monolayer corresponds to ~(1–2)·10^15^ atoms per cm^2^, then when the degree of coverage changes from 0.003 ML to 0.01 ML, the concentration of rhodium atoms on the In_2_O_3_ surface will change in the range from (3–6)·10^12^ to (1–2)·10^13^ at/cm^2^. This means that with a coating of 0.01 ML of Rh on the surface of one In_2_O_3_ crystallite with a size of 10 nm, there can be up to 10–20 isolated rhodium atoms. As we indicated earlier, the process of sensor response increase stops when the formation of 3D clusters begins. It occurs due to the coalescence of rhodium atoms located on the In_2_O_3_ surface. Simple calculations show that with a coverage of 0.1 ML of Rh and a crystallite size of 10 nm, on the surface of one crystallite there can be between two to eight clusters with a size of 1 nm. In the calculations, we took into account the size of rhodium atoms equal to 0.268–0.346 nm (empirical—calculated). It should be noted that the transition from an atomically dispersed state to a cluster state at a certain degree of surface coverage with atoms of noble metals, including rhodium, was confirmed experimentally in many works [63,64]. For example, as applied to Pt atoms, it was found that in order to maintain the atomically dispersed state on the Fe_2_O_3_ surface, it is necessary to maintain a very low density of Pt atoms (<0.1 wt%) [64]. In this case, the atomically dispersed state is retained even in reducing environments at elevated temperatures. 

However, the situation discussed above (Equation (1)) in such an ideal case is realized for sensors operated only in dry atmosphere. For sensors operated in usual atmosphere at temperatures of 200–250 °C, which are optimal for ozone sensor operation, hydroxyl groups predominate on the surface of metal oxides [62,65,66]. It was found that at these operating temperatures the dissociative adsorption of water molecules at the surface of n-type semiconductor metal oxides is energetically more preferable than dissociative adsorption of oxygen, and as a result, the surface becomes hydroxylated [65]. Therefore, without any doubt, when detecting ozone in a humid atmosphere, OH-groups must participate in the ozone detection reaction. It is their participation, according to Korotcenkov et al. [31], which causes an increase in the response time of sensors in comparison to the process of ozone detection in a dry atmosphere (see Figure S4, supplementary materials). Several possible reactions involving ozone and OH-groups can be proposed, which may be accompanied by an increase in the concentration of chemisorbed oxygen-containing species on the surface of In_2_O_3_. However, all these reactions involve the formation of intermediate ${\mathrm{HO}}_{X}^{-}$ surface-bound radicals such as ${\mathrm{HO}}_{2}^{-}$ or ${\mathrm{HO}}_{3}^{-}$ (see Figure 3b). Their appearance upon interaction of water and OH radicals with ozone has been repeatedly proved experimentally and theoretically [67,68,69,70]. On the surface of metal oxides, ozone also intensively interacts with surface hydroxyl groups. IR spectroscopy performed by Bulanin et al. [71] showed that after the interaction of ozone with a hydrated surface of TiO_2_, the intensity of the band at 3751.5 cm^−1^, corresponding to OH-groups, decreases and a strong and wide band appears at about 3670 cm^−1^, belonging to perturbed hydroxyls. If the amount of adsorbed ozone is large enough, then the band of free OH groups disappears completely, while the maximum of the band of perturbed hydroxyls shifts to 3640 cm^−1^. It is important to note here that oxygen adsorption did not lead to such a strong disturbance of hydroxyls.

As for the reasons for the decrease in the resistance of Rh/In_2_O_3_ films in ozone atmosphere taking place in the region of cluster state of rhodium (Rh coverage on the In_2_O_3_ surface greater than 0.01 ML), and the decrease in the sensor response to ozone, which is associated with this effect, here we are forced to state that these changes are a consequence of reduction of negative charge captured by surface species adsorbed on the surface of metal oxide crystallites during ozone detection. This is possible only if 3D clusters of rhodium on the surface of In_2_O_3_ block the centers of dissociative ozone adsorption of the surface of In_2_O_3_ or form a channel of ozone decomposition, which occurs on the surface of a rhodium cluster without any electronic exchange between the rhodium cluster and the bulk of the metal oxide. Due to the appearance of an additional channel for ozone dissociation, the amount of ozone directly interacting with the In_2_O_3_ surface decreases. The latter is more likely from our point of view. A similar approach to explain the observed characteristics of surface doped Pt/SnO_2_ gas sensors was used by Degler et al. [19]. They suggested that the decrease in the sensitivity of SnO_2_-based sensors with atomically distributed Pt to CO in a dry atmosphere is a consequence of the appearance of a separate Pt oxide phase on the SnO_2_ surface, which offers additional reaction sites for CO oxidation, which are not electronically coupled to the SnO_2_. In other words, the decreased sensor signals in dry air are caused by a parallel reaction of CO oxidation on the PtO_2_ phase with no effect on the electrical properties of SnO_2_.

With regard to the not so pronounced decrease in the sensor response of HT films of In_2_O_3_ with a Rh coverage more than 0.01 ML as compared to LT films, this behavior can be explained as follows. Due to the more intense coalescence of rhodium on the HT In_2_O_3_ surface and the formation of larger 3D clusters, their density on the In_2_O_3_ surface decreases. This process reduces the filtering ability of the rhodium clusters to prevent the interaction of ozone with the metal oxide surface. According to [21], the transition from the atomically dispersed state to 2D and subsequently to the 3D cluster state of Rh on the In_2_O_3_ surface causes the deviation of the dependence of the I(Rh3d)/I(In3d) ratio on the surface coverage by rhodium from the linearity. Here, I(Rh3d) and I(In3d) are the intensities of Rh3d and In3d XPS peaks. During this transition, the area occupied by rhodium atoms does not increase in proportion to the amount of Rh deposited on the In_2_O_3_ surface. The larger the size of 3D clusters formed during metal deposition, the greater the deviation from the dependence constructed under the assumption of the atomically dispersed state of the deposited atoms should be. As one can see in Figure S5 (supplementary materials), the greatest deviation from the linearity of the indicated dependence is observed for HT In_2_O_3_ films.

**Scheme 1:**
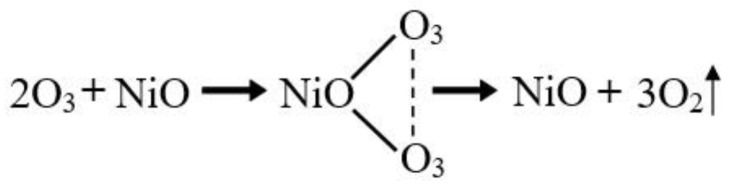
Ozone decomposition by molecular mechanism.

**Scheme 2:**
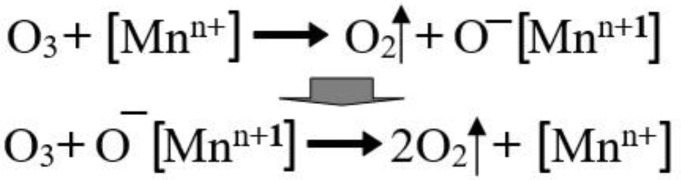
Ozone decomposition with participation of atomic oxygen.

It should be noted that our approach to explaining the results obtained is in good agreement with many of the previously published results [5,13,22,72,73]. For example, it has been reported that trace amounts of Rh (0.005 wt.%, which corresponds to the atomic dispersion of the Rh) can promote direct participation of lattice oxygen of metal oxides (NiO, CeO_2_–TiO_2_) including even chemically very inert supports such as aluminum oxide (Al_2_O_3_), to oxidize reactant molecules such as methane, while the fundamental mechanism is elusive [13]. Karin et al. [13] believe that the preferable Rh–O rather than the reductive Rh–Me bond formation together with the ability of Rh to accumulate the large amounts of electrons are crucial factors to drive the unique reactions promoted by a single atom of Rh. At the same time, the mechanisms described by us in Figure 3c,d were used to explain the decomposition of ozone in the presence of nickel oxide (NiO) [73] and manganese dioxide (MnO_2_) [22]. It was found that part of the ozone was decomposed by molecular mechanism (Scheme 1), and the other with the participation of atomic oxygen (Scheme 2), which appears due to the dissociation of ozone into atomic oxygen species:

It should be noted that the reactions shown in Figure 3c,d cannot be implemented on a single atom of Rh, since these reaction pathways require two nearby active metal atoms, which is impossible in this case. The results obtained by Beyer et al. [4] are also in good agreement with our conclusion. They showed that Rh/MeO_x_ catalysts with relatively large rhodium particles (*d*_m_ = 2.1–2.4 nm) exhibited significantly higher activity at decomposition of N_2_O at low reaction temperatures (200–300 °C) as compared to catalysts with smaller rhodium particles (*d*_m_ = 1.0–1.4 nm). It should also be borne in mind here that if on the surface of In_2_O_3_ modified with Rh the dissociation of ozone occurs according to Scheme 2, then in this case electronic exchange between the Rh cluster and the bulk of the metal oxide is possible. This means that with a more detailed examination of the processes occurring on the surface of In_2_O_3_ modified with Rh clusters, it will be necessary to take into account the contribution of both electronic and chemical properties and processes.

**Figure 4:**
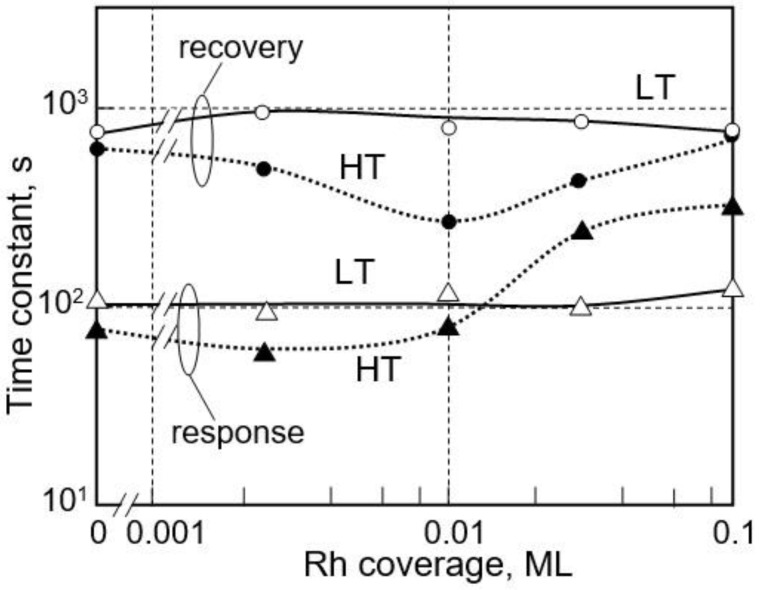
Effect of surface modification of LT and HT In_2_O_3_ films with rhodium on the time constants of the sensor response to ozone (~1 ppm). Response time (τ_res_) and recovery time (τ_rec_) were measured at 225 °C.

The results of the analysis of the kinetics of sensor response are shown in Figure 4. It can be seen that while for LT films the response and recovery times are practically independent of the surface modification with rhodium, for HT In_2_O_3_ films, the effect of rhodium on the kinetics of the sensor response is observed. We are not yet ready to offer a detailed explanation of the results obtained. More detailed research is required. In [74], an attempt was made to analyze the cross-sensitivity of In_2_O_3_ to O_2_, O_3_ and H_2_O using XPS measurements. However, the conditions for carrying out these experiments are far from the real conditions used in the operation of gas sensors. Currently we can only state that for HT films the main changes take place in the region of ~0.01 ML of Rh. This confirms our hypothesis that under such Rh coverage there is a change in the mechanism of the processes responsible for the sensor response.

## 4. Conclusions

Studies have shown that the size effect has a significant influence on the activity of noble metals used to optimize the parameters of gas sensors, and this factor should be taken into account when developing a technology for their manufacture. In particular, when rhodium is used to modify the surface of In_2_O_3_ films designed for application in gas sensor aimed for ozone detection, the effect achieved during such modification has a strong size-dependent nature. If at low coverages (<0.01 ML of Rh) it is possible to achieve an increase in the sensor response to ozone, then at large coverages (>0.01 ML of Rh), modification of the In_2_O_3_ surface with rhodium will lead to a decrease in the sensor response. This effect is associated with the transition from the atomically dispersed state of rhodium on the surface of In_2_O_3_ to the 3D cluster state with a corresponding change in the mechanism of interaction of Rh with ozone and In_2_O_3_.

## Data Availability

Data is contained within the article and supplementary material. Additional data available on request.

## Supplementary Materials

The following are available online at https://www.mdpi.com/1424-8220/21/5/1886/s1, Figure S1: Operation temperature influence on conductivity response of undoped In_2_O_3_-based sensors, Figure S2: Characterization of In_2_O_3_ films, Figure S3: The effect of Rh deposition on binding energies of Rh3d5/2 and C1s, Figure S4: Influence the air humidity on temperature dependencies of response and recovery times, Figure S5: The effect of rhodium coverage on the intensity of XRS Rh3d5/2 peaks.
