# Supplementary material for: Ozone Sensing by In2O3 Films Modified with Rh: Dimension Effect
Source: Sensors (Basel). 2021 Mar 8;21(5):1886. doi: 10.3390/s21051886 (PMC7962652; doi:10.3390/s21051886)
Supplement: Supplementary file 1 [file sensors-21-01886-s001.pdf]

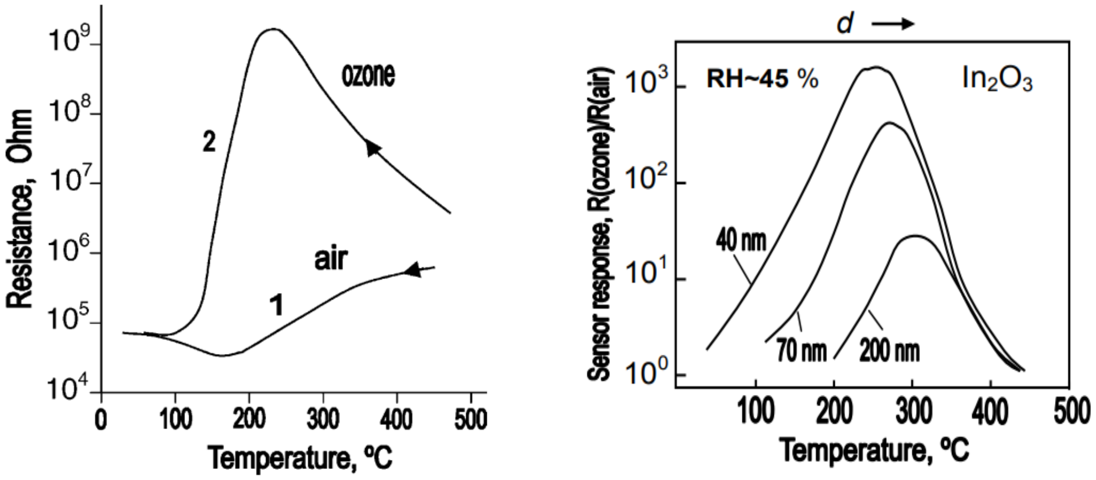

(a) **(b)**

Figure S1. (a) Temperature dependencies of the In_2O_3 film resistance during ozone detection (T_{pyr} = 475 °C; d ~ 40 nm): (1) air; (2) air + ozone (1 ppm); (b) Film thickness influence on temperature dependences of In_2O_3-based sensors response to ozone. T_{pyr}= 475 °C. More detail information one can find in [28, 29, 40].

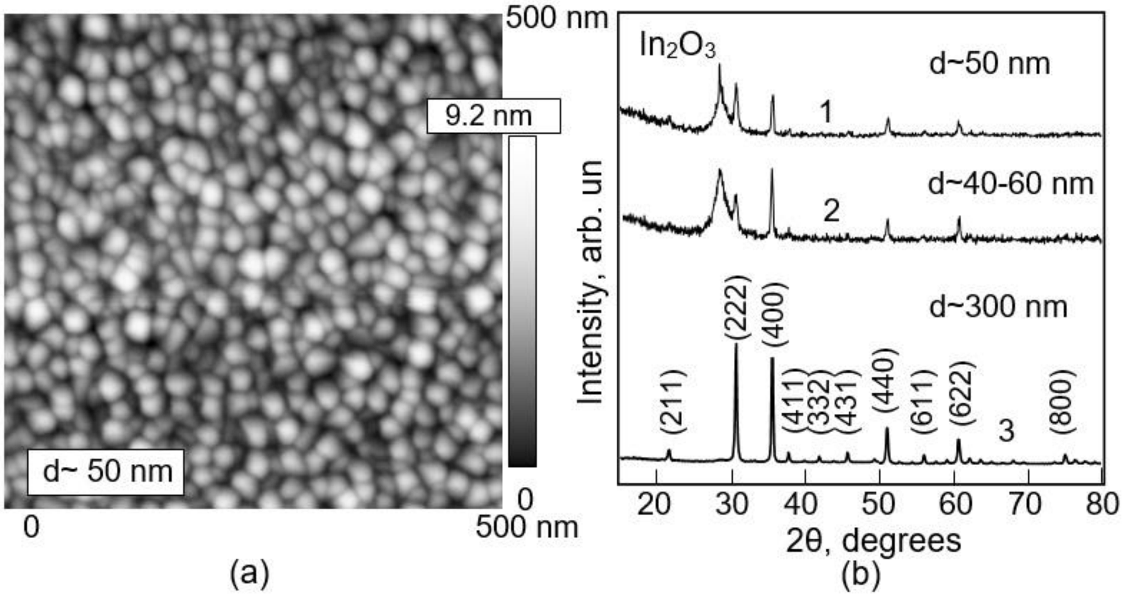

Figure S2. (a) - Typical AFM image of thin In_2O_3 films deposited by spray pyrolysis at T_{pyr} =450-475 °C; (b) XRD patterns and its Miller indices on each diffraction peak for In_2O_3 films deposited in different conditions: 1- *d*~50 nm, T_{pyr} =350-400 °C; 2 - 1- *d*~40-60 nm, T_{pyr} =450-500 °C; 3 - 1- *d*~300 nm, T_{pyr}=400-425 °C. [21].

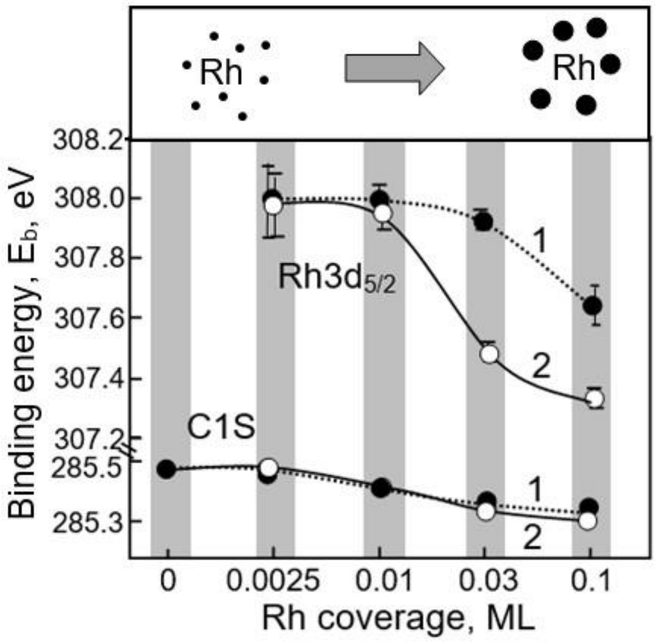

Figure S3. The effect of Rh deposition on the In_2O_3 surface on binding energies of Rh3d5/2 and C1s. 1 - LT In_2O_3 films; 2 - HT In_2O_3 films. X-ray photoelectron spectroscopy (XPS) experiments were performed by using an Omicron EA 125 multichannel hemispherical analyzer with Mg Kα line (1253.6 eV) as a primary photon source. The photoelectron spectra of In3d, O1s, C1s, and Rh3d core levels were acquired at normal emission of the photoelectrons with respect to the sample surface. The measurement methodology and the results obtained during XPS study of Rh/In_2O_3 films are described in more detail in [21].

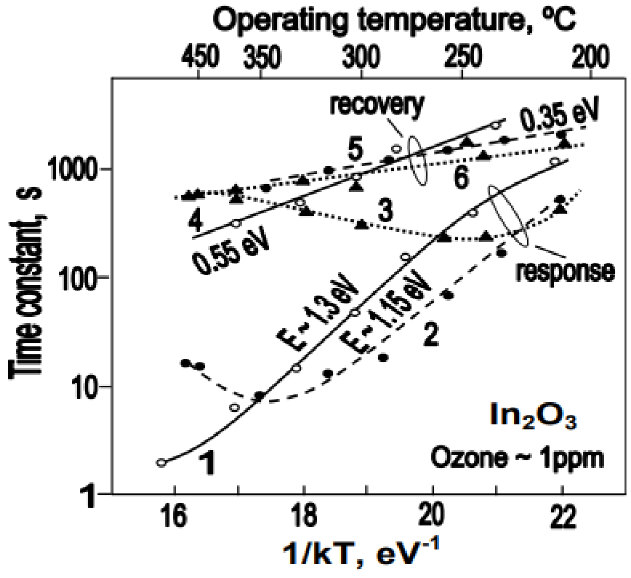

Figure S4. Influence the air humidity on temperature dependencies of (1-3) response and (4-6) recovery times during ozone detection by In_2O_3 -based sensors: (1,4)-~0.5% RH; (2,5)-~25-30 RH; (3,6)-~60%RH; T_{pyr}= 475 °C; d~200 nm; 1.0 M $InCl_3$-solution. More detail information regarding kinetics of conductivity response of In_2O_3-based sensors one can find in [29, 31, 40, 41].

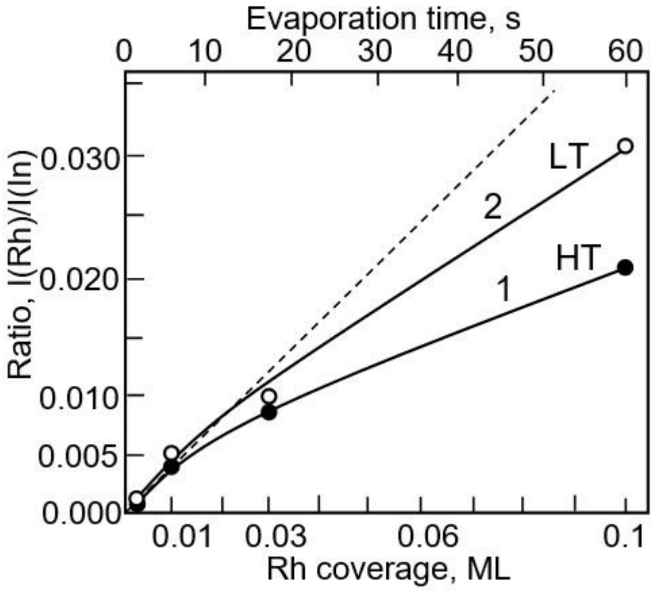

Figure S5. The effect of rhodium coverage on the intensity of XRS Rh3d5/2 peaks normalized to the intensity of XRS In3d5/2 peaks: 1 - HT In_2O_3 films; 2 - LT In_2O_3 films. [21].
